# The Use of V.A.C. VERAFLO CLEANSE CHOICE in the Burn Population

**DOI:** 10.7759/cureus.3632

**Published:** 2018-11-26

**Authors:** Marc R Matthews, Aaron Hechtman, Asia N Quan, Kevin N Foster, Luis G Fernandez

**Affiliations:** 1 Surgery, Arizona Burn Center, Phoenix, USA; 2 Pharmacy, Maricopa Integrated Health System, Phoenix, USA; 3 Surgery, University of North Texas Health Science Center, Fort Worth, USA

**Keywords:** burn, cleanse choice, hypochlorous acid

## Abstract

Negative pressure wound therapy (NPWT) is routinely used in the treatment of acute and chronic wounds. The technology continues to evolve with improved results NPWT is routinely used at the Arizona Burn Center and the addition of the V.A.C. VERAFLO CLEANSE CHOICE^TM^ with its reticulated open foam device has been used with promising results in a variety of complicated wounds. We present a case series involving the use of this negative pressure wound therapy device and irrigation in burn and necrotizing soft tissue patients treated at the Arizona Burn Center.

## Introduction

Contact burns are very prevalent during the Arizona summer [1]. Negative pressure wound therapy (NPWT) has been effective in the treatment of acute and chronic wounds. NPWT is routinely used to assist in the formation of granulation tissue, increase in wound blood flow, removal of bacteria and its fibrinous exudate, wound edema, and subsequent wound closure [2]. Advances in this technology have allowed for improved results utilizing new devices, such as V.A.C. VERAFLO CLEANSE CHOICE™ (VVCC, KCI, Acelity, San Antonio, TX, US). VVCC is a reticulated open cell foam dressing (ROCF) that allows for the instillation of an irrigant in order to lavage the wound bed with the removal of the thickened wound exudate and prokaryotic materials. The VVCC also helps large and complex acute and chronic wounds not only remove fibrinous and bacterial exudate but also increases granulation tissue [3-4]. The use of instillation therapy with various irrigants (normal saline, prontosan, and hypochlorous (HOCl) acid) have also been described in combination with NPWT and ROCF dressings [3-5]. Such new adjuncts have helped with a more rapid tissue defect contraction, bacterial exudate clearance, and subsequent skin closure with skin grafting techniques. The addition of Vashe® (Stead Med, Inc, Fort Worth, TX, US) (HOCl) instillation therapy provides an effective medium to assist in a neutrophil respiratory burst in the killing of bacteria thereby eliminating any prokaryotic organisms within 15 seconds of contact. At the Arizona Burn Center, we routinely use VVCC for large soft tissue defects following deep skin tangential excisions or soft tissue debridements whether from burns or necrotizing soft tissue infections. Typically, requiring multiple NPWT changes over weeks to allow for defect closure prior to skin grafting, VVCC has been trialed at our burn center with encouraging results. The purpose of this case series is to review the use and promising results of VVCC and HOCl irrigation in the treatment of a variety of complicated wounds at our burn center.

## Case presentation

Case 1: contact burn to left hip

**Figure 1:**
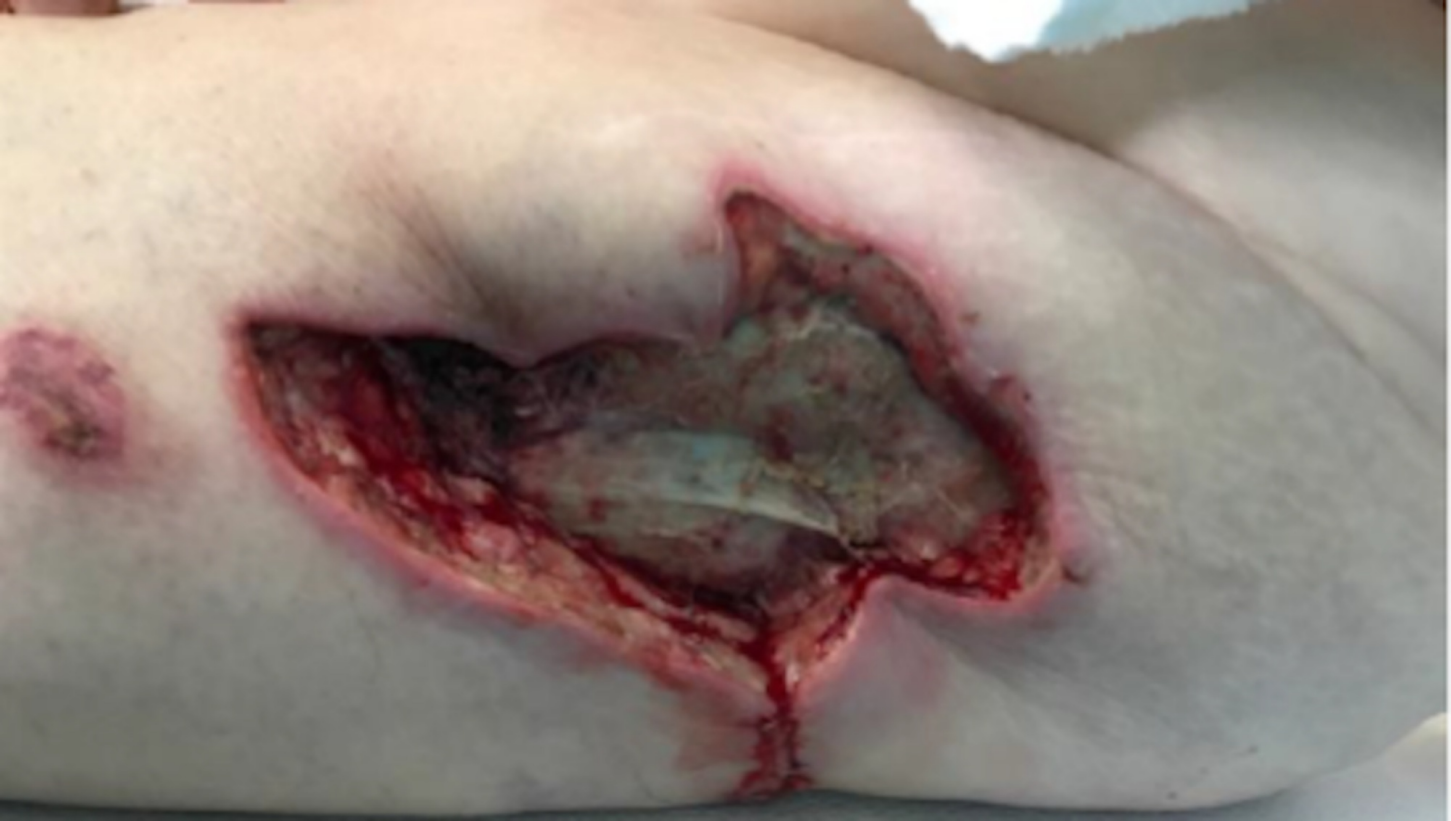
Initial debridement of burn eschar revealing deep necrotic tissue still in place

**Figure 2:**
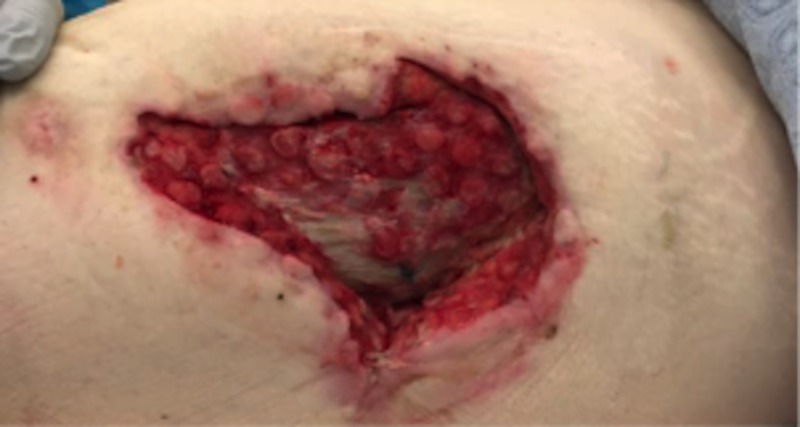
Rapid improvement in wound granulation tissue showing classic VVCC “comedone” development with increased granulation tissue within the ROCF hole boundaries VVCC: V.A.C. VERAFLO CLEANSE CHOICE™; ROCF: reticulated open cell foam dressing

A 77-year-old male was admitted for contact burns to the bilateral lower extremities and over the left hip, involving not only the skin but a burn injury down to the fascia and muscle. The patient had multiple medical comorbidities, including diabetes mellitus and hyperlipidemia. After debridement and dressing changes (Figure 1), attempts at grafting the site with split-thickness skin grafts (meshed 2:1, 150 sq cm) resulted in graft loss within days, secondary to an inadequate depth of debridement and the lack of appropriate granulation tissue presence. Therefore, the patient was transitioned into VVCC NPWT to assist in irrigation debridement, granulation tissue formation, and wound contraction with the addition of Vashe® instillation at 30 ml for 20 minutes duration every three hours before returning to suction for over two weeks. NPWT suction was maintained at 125 mmHg while on suction. Rapid improvement in wound granulation tissue formation was noted (“comedones” or discrete and increased granulation tissue within the ROCF hole boundaries, Figure 2). The patient continues to follow up in our clinic with planned skin grafting at the time of this manuscript’s submission.

Case 2: contact burn to left buttock

**Figure 3:**
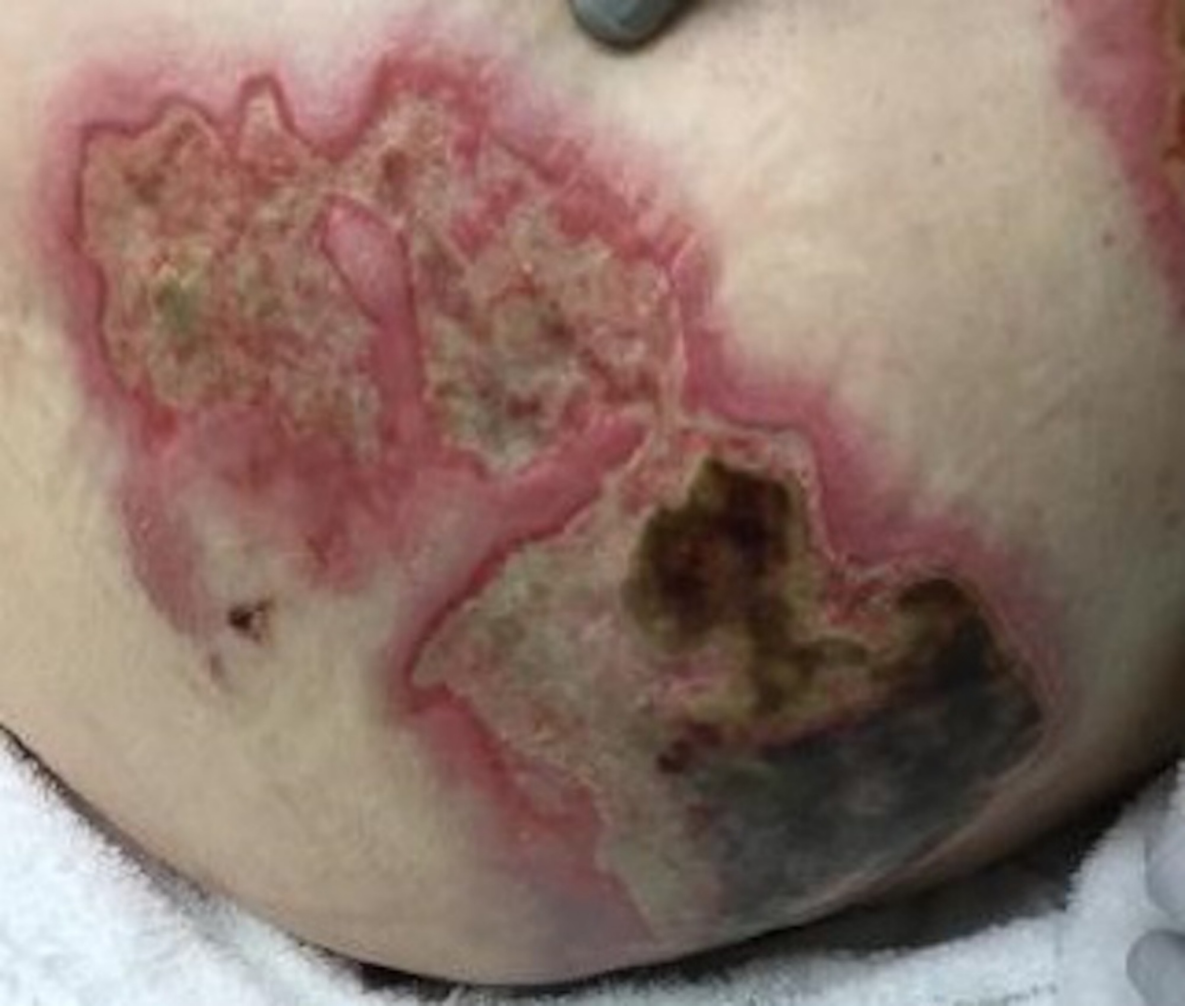
Initial third-degree burn wounds to the patient’s left buttocks and hip

**Figure 4:**
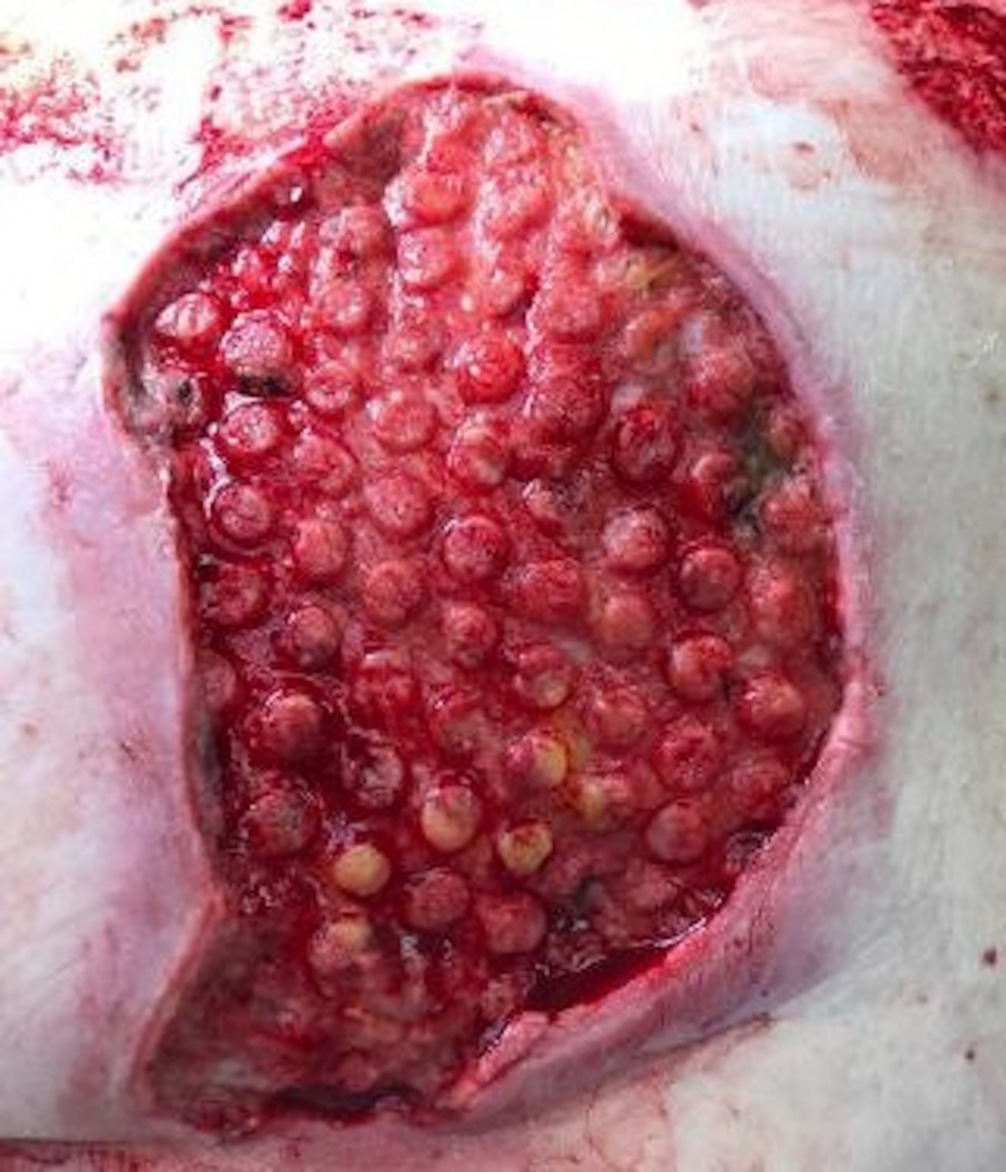
Rapid improvement in wound granulation tissue after several days post-excision showing classic VVCC “comedone” development with increased granulation tissue within the ROCF hole boundaries VVCC: V.A.C. VERAFLO CLEANSE CHOICE™; ROCF: reticulated open cell foam dressing

**Figure 5:**
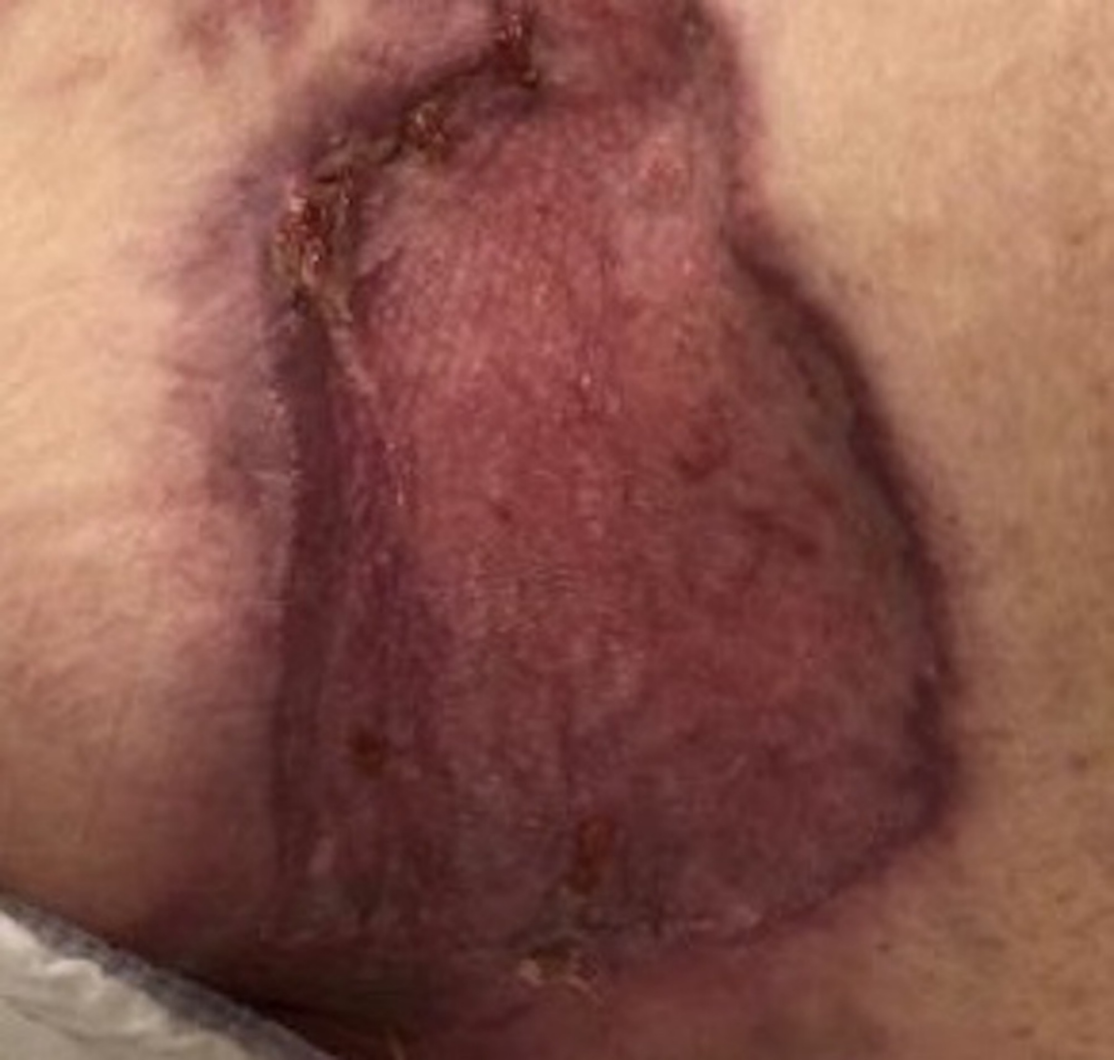
Closure of left buttock and hip wounds after standard split-thickness skin grafting techniques

An 87-year-old male was admitted for contact burns following a syncopal episode. The wounds were full thickness burns requiring excision down to the subcutaneous tissue (Figure 3). A significant soft tissue defect over the left buttock was noted and VVCC NPWT was placed over the wound. Vashe instillation was started at 30 ml for 20 minutes every three hours before returning to NPWT suction at 125 mmHg, which was applied to assist in the debridement and granulation of the wound. Short-term therapy with the VVCC resulted in a healthy granulation bed demonstrating near-skin-level comedone granulation tissue formation (Figure 4). The patient received an autologous skin graft (meshed 1:1, 200 sq cm) after the completion of VVCC therapy with 100% skin graft take (Figure 5).

Case 3: contact burns to bilateral buttocks

**Figure 6:**
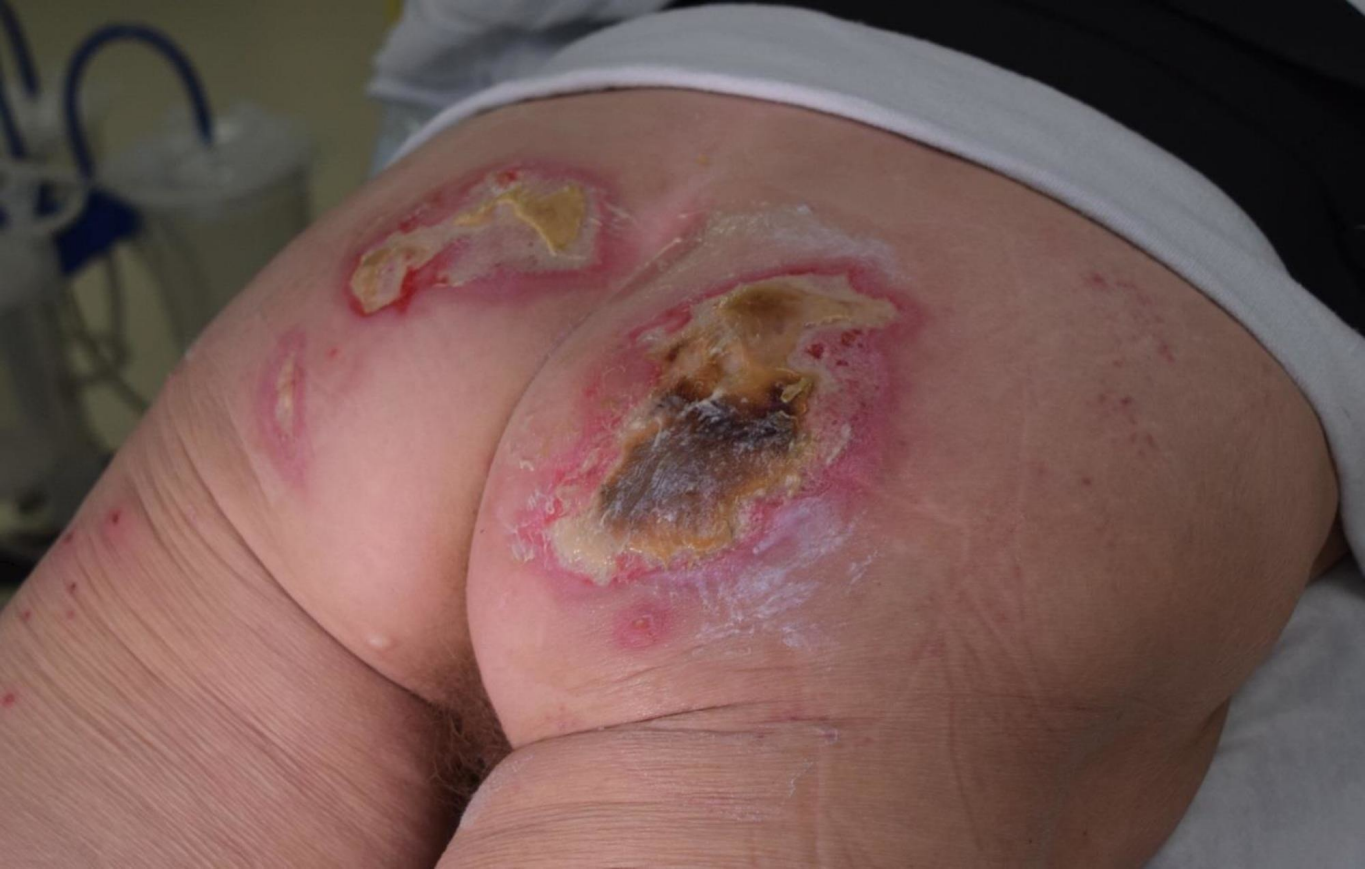
Initial bilateral buttock contact burn wounds

**Figure 7:**
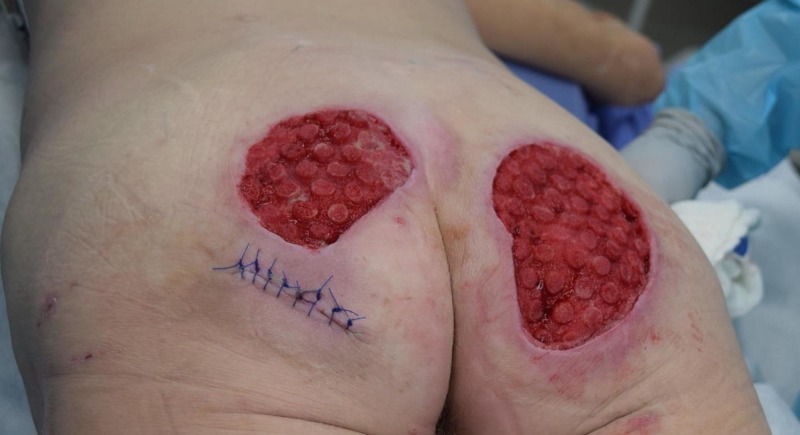
Afterburn wound eschar debridement and several days of VVCC, appearance of the classic VVCC “comedone” development with increased granulation tissue within the ROCF hole boundaries VVCC: V.A.C. VERAFLO CLEANSE CHOICE™; ROCF: reticulated open cell foam dressing

**Figure 8:**
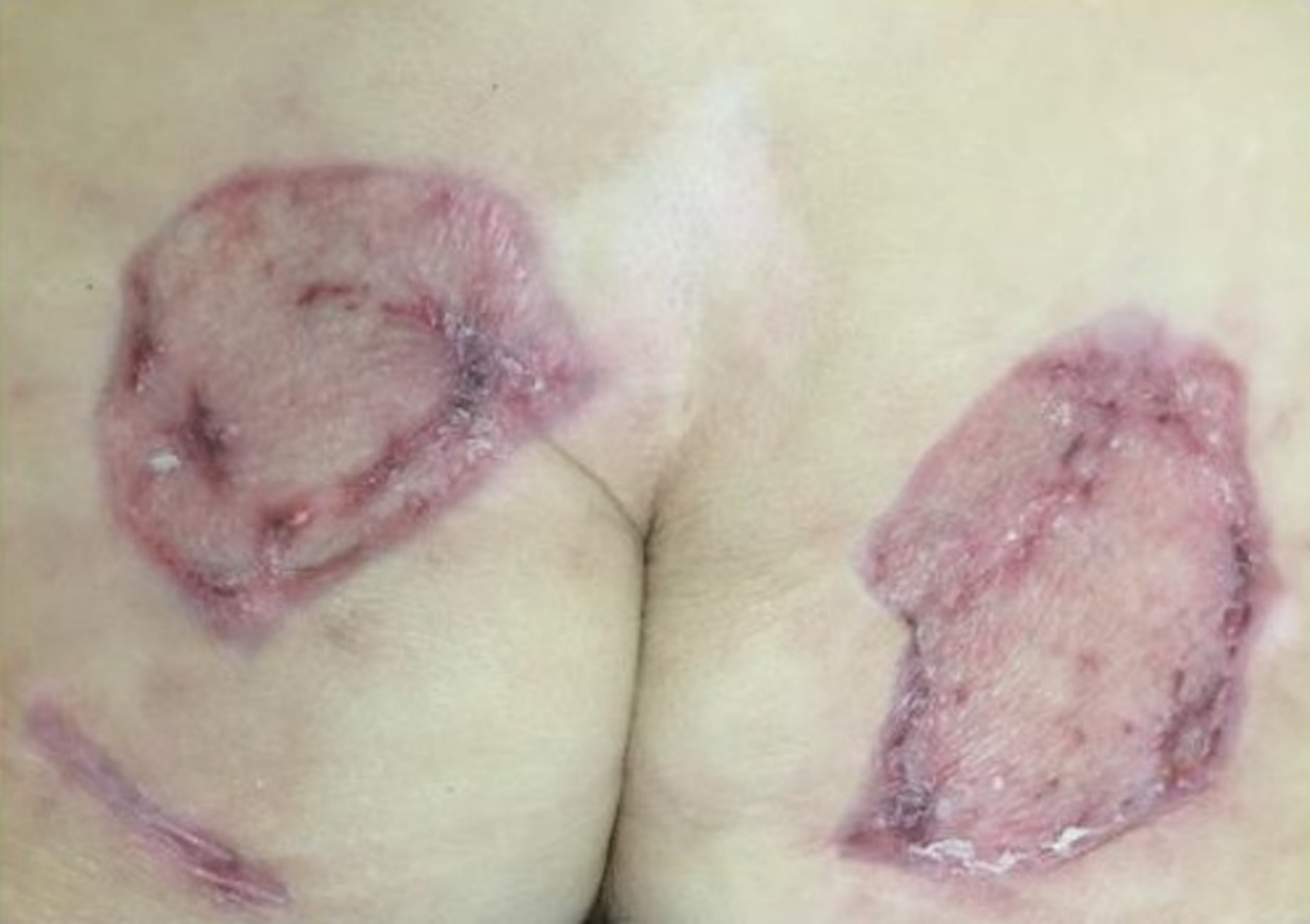
Closure of the bilateral buttock wounds after standard split-thickness skin grafting techniques

A 61-year-old female was admitted with full thickness contact burns to the bilateral buttocks (Figure 6). Initially, this was debrided down to the soft tissues. After a week of dressing changes, an autologous skin graft was applied (meshed 1:1, 100 sq cm) but the skin graft failed and necessitated a return to the operating room for further debridement. A VVCC NPWT was placed with improved granulation tissue formation (Figure 7). Vashe solution was used to irrigate the wound with 30 ml for a 20 minute dwell time every three hours with a NPWT suction of 125 mmHg. Eventual split-thickness skin grafting to the bilateral buttocks had a 100% graft take (Figure 8).

Case 4: contact burns to the anterior torso and bilateral lower extremities

**Figure 9:**
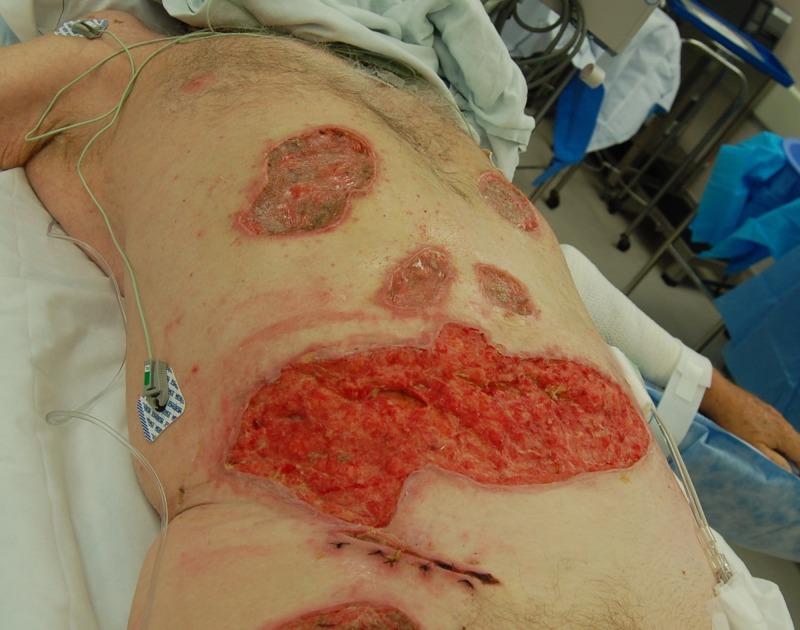
Initial debridement of an abdominal burn eschar

**Figure 10:**
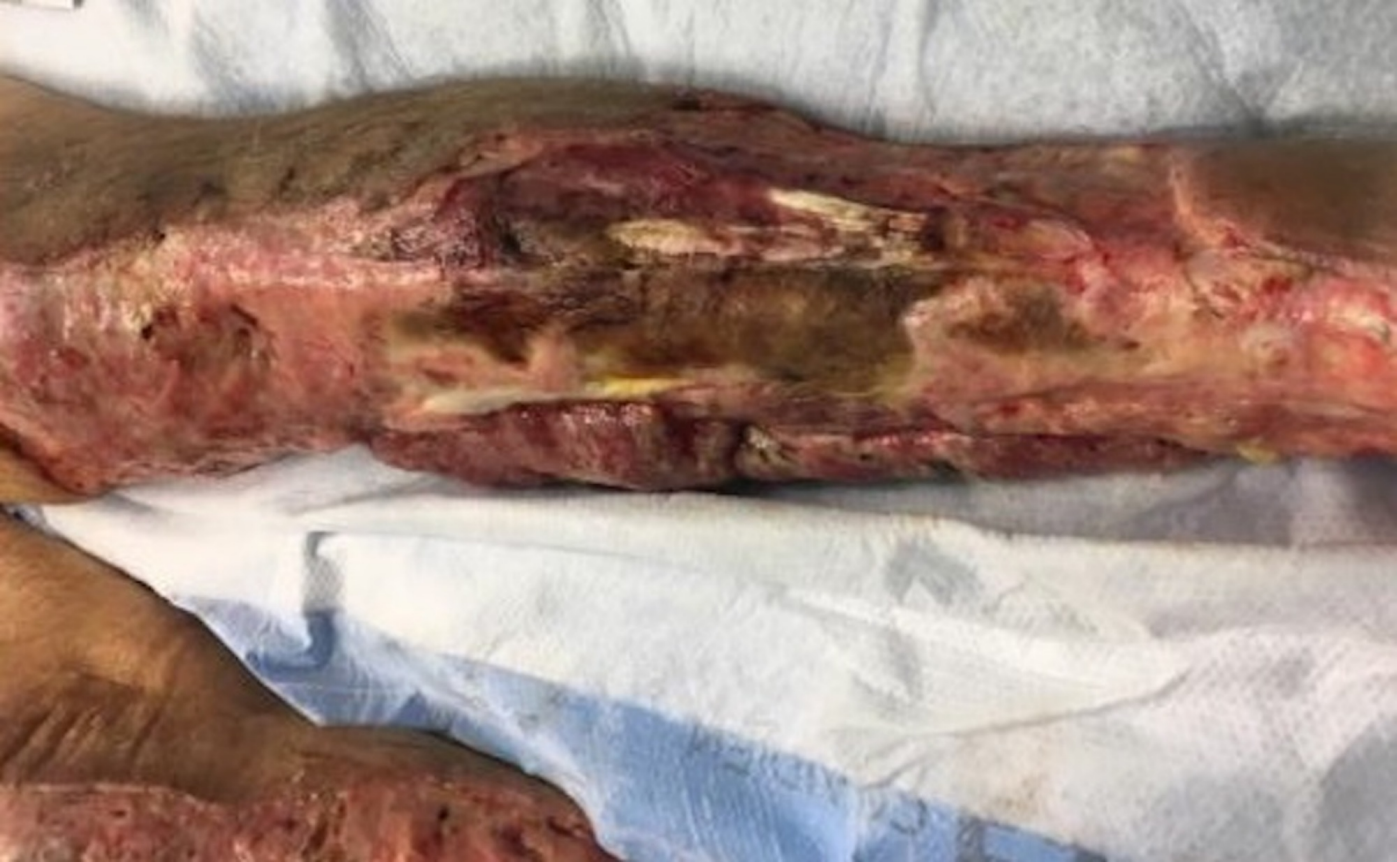
Initial left leg burn eschar

**Figure 11:**
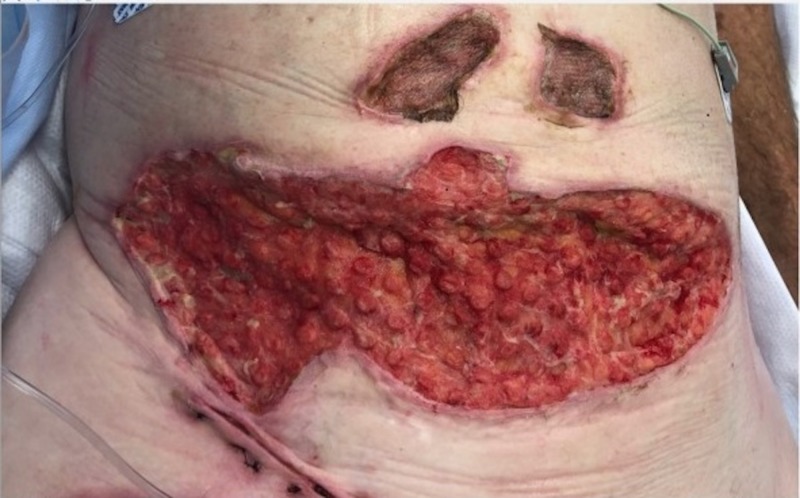
Followed by the appearance of the classic VVCC comedones granulation tissue VVCC: V.A.C. VERAFLO CLEANSE CHOICE™

**Figure 12:**
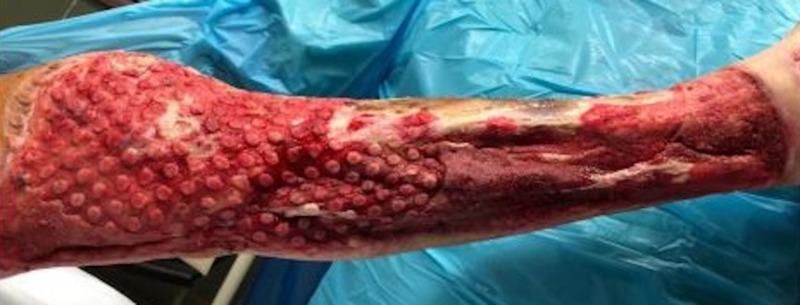
Followed by the appearance of the classic VVCC comedones granulation tissue after the multiple debridements and applications of the VVCC device VVCC: V.A.C. VERAFLO CLEANSE CHOICE™

**Figure 13:**
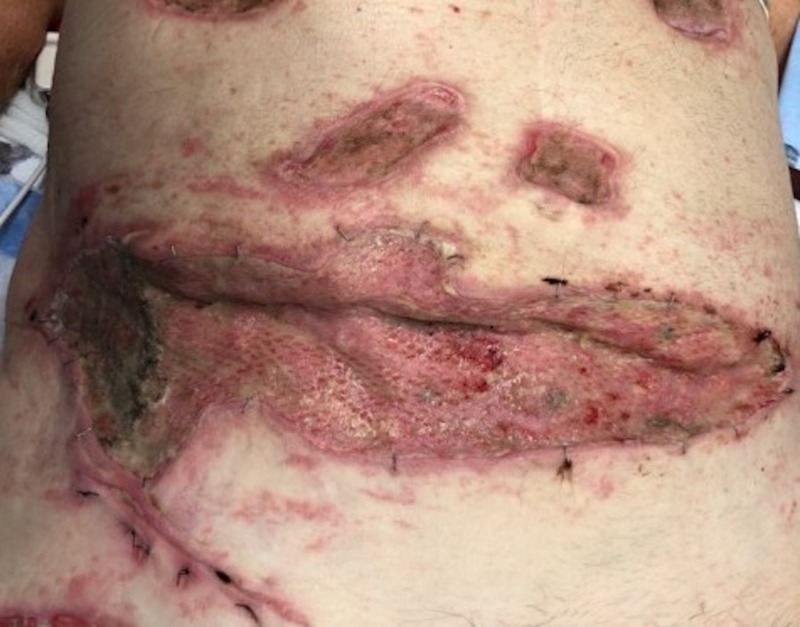
Finally, closure of the abdominal wound with split-thickness skin grafts

**Figure 14:**
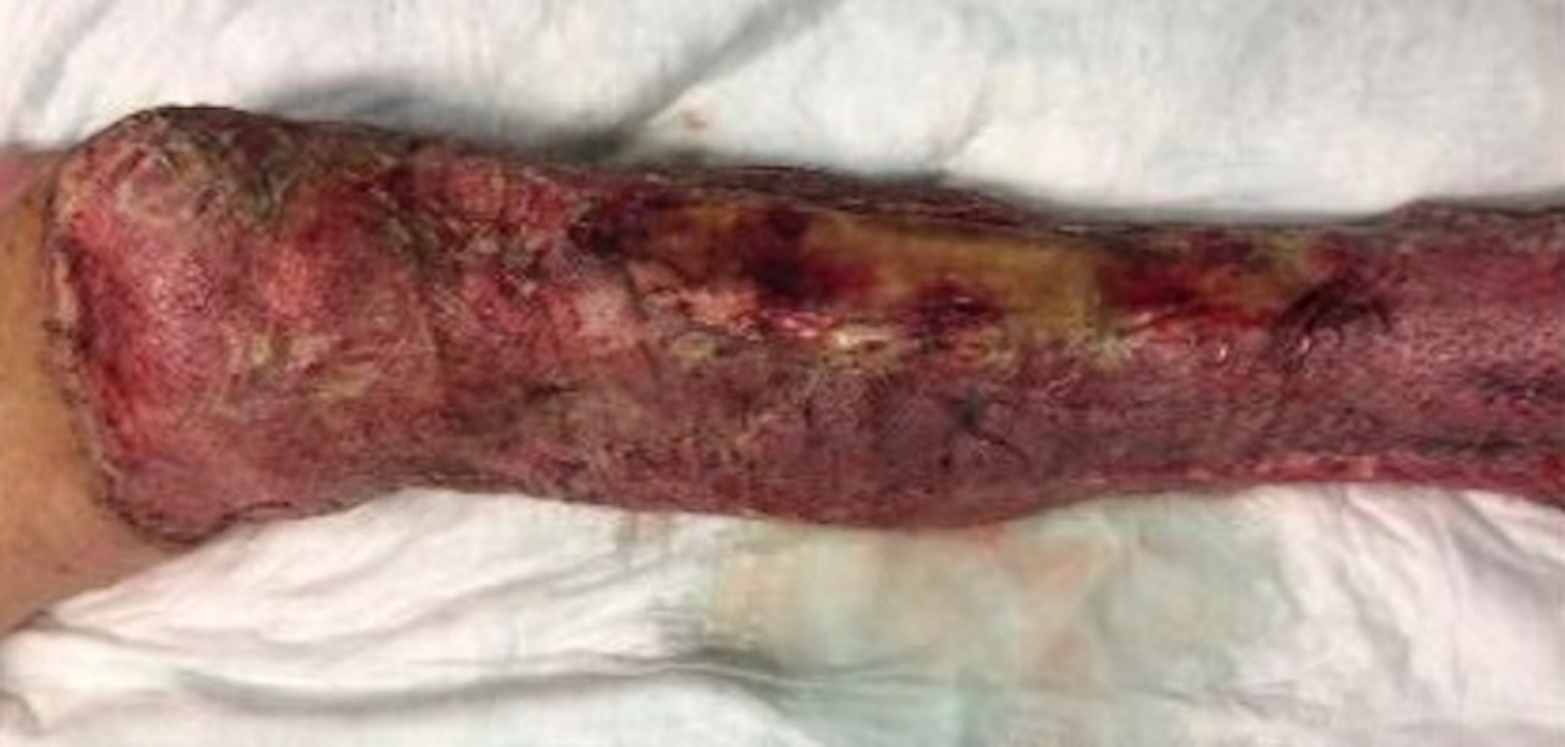
Subsequent closure of the left leg wound with split-thickness skin grafts

A 76-year-old male admitted with 21% total body surface area (TBSA) full thickness contact burns to his anterior torso and bilateral lower extremities following a ground level fall. The patient was debrided multiple times, which included a right, above-knee amputation, and autologous skin grafting to the anterior upper torso and right thigh. Deeper tissue defects of the lower abdomen (Figure 9) and left lower extremity (Figure 10) required the placement of the VVCC NPWT system with HOCl instillation of approximately 30 ml to the lower abdomen and 30 ml to the left lower extremity for 30 minutes every three hours with an NPWT suction of 125 mmHg. After two weeks of therapy (Figures 11-12), the patient had an autologous skin graft applied to the anterior torso and left lower extremity (meshed 2:1, 1400 sq cm). There was a 100% skin graft take to the lower abdominal torso and 90% skin graft take of the left lower extremity (Figures 13-14).

Case 5: necrotizing fasciitis of the lower abdominal wall

**Figure 15:**
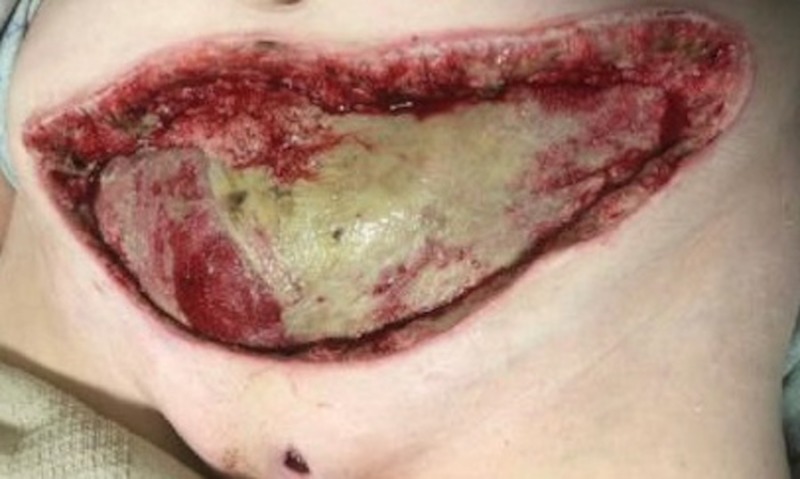
Large anterior abdominal wound after serial debridements

**Figure 16:**
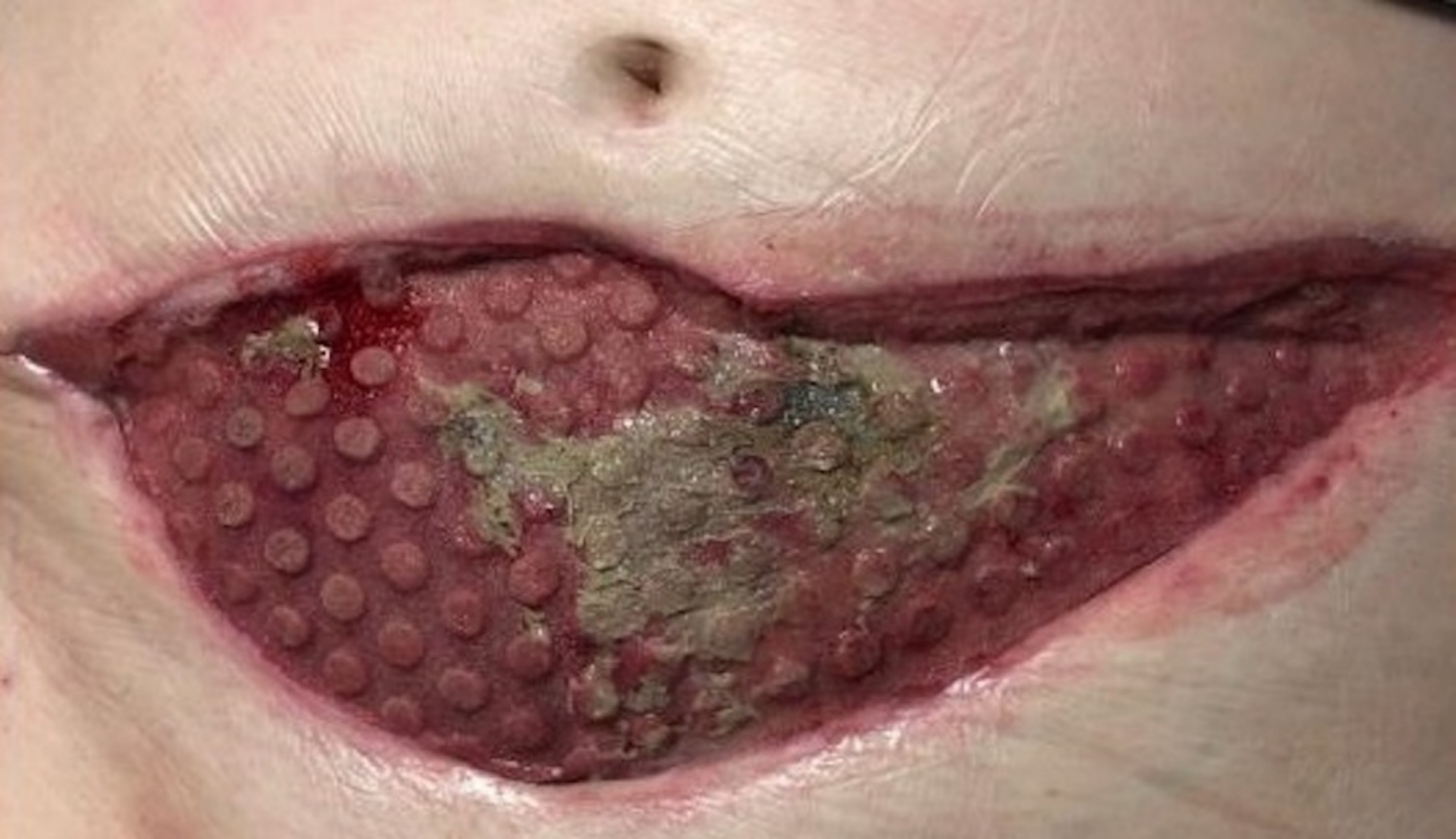
Contraction of the large anterior abdominal wound and development of comedone granulation tissue around a small area of remaining fibrinous exudate

**Figure 17:**
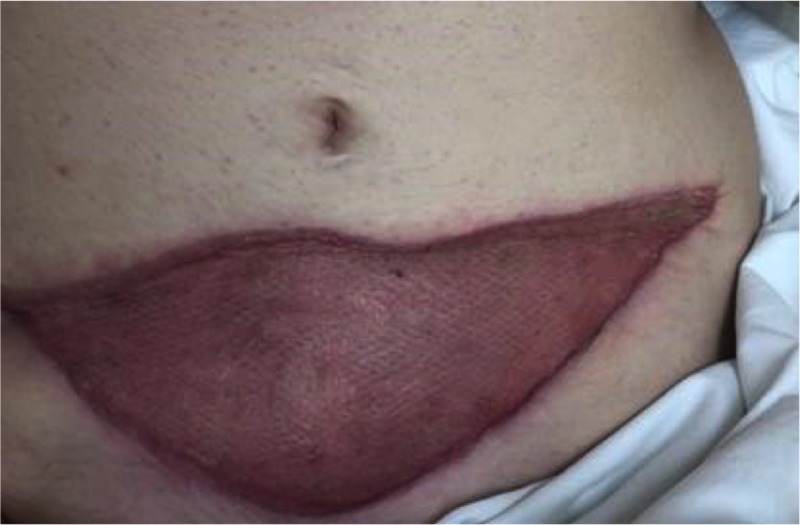
Lower abdomen following completion of therapy and skin grafting

A 55-year-old female was admitted with a past medical history of multiple medical comorbidities, including diabetes mellitus (type 1), rheumatoid arthritis requiring immunosuppressant therapy, and baseline liver dysfunction. She had an abdominal wall necrotizing fasciitis due to an infected subcutaneous insulin pump. The patient underwent extensive debridement down to the rectus fascia and was left with a large soft tissue defect (Figure 15). The patient had placement of the VVCC NPET once the wound was debrided to viable tissue and the initial infection was controlled. The wound has been granulating and contracting with V.A.C. VERAFLO CLEANSE CHOICE™ also utilizing HOCl instillation of 30 ml with a dwell time of 20 minutes every three hours and then returning to a negative pressure of 125 mmHg. Because of the patient’s multiple medical issues and compromised immune system that would normally impair wound healing, the patient is weeks away from wound closure (Figure 16). The patient's therapy was completed with the closure of the wound following split-thickness skin grafting (Figure 17).

## Discussion

The use of the V.A.C. VERAFLO CLEANSE CHOICE™ (VVCC) has advanced our wound therapy regimen in the burn population and those with large wounds such as necrotizing fasciitis. Some burn center patients have large soft tissue defects following debridement that only require porcine xenografting or cadaveric allografting before they undergo split-thickness skin grafting while other patients have much deeper defects that require a healthy granulation bed before ultimately undergoing closure. The VVCC has provided an opportunity to effectively improve complex wound healing by the better elimination of the necrotic material and faster generation of granulation tissue in conjunction with the use of HOCl solution as reported by Fernandez and others [5-6]. Heibert and Robson described the reduction in bacterial growth with the use of HOCl acid compared to normal saline in the treatment of infected wounds [7]. In addition, it has been shown that the use of HOCl has led to a decrease in bacterial growth, and that even at low concentrations of HOCl, bacterial growth and cell division are decreased [8]. Studies in vitro have even shown that the presence of a prokaryotic biofilm is disrupted by at least 90% with just a brief exposure to HOCl [9]. When HOCl is used with an NPWT device, it has led to the more rapid development of granulation tissue and healthier wound beds [10]. Irrigation with HOCL should now be considered instrumental when the ROCF NPWT is used and irrigation is required to further treat the heavily exudative wounds or wounds that have heavy prokaryotic contamination before any attempt at closure. Other studies have also shown the benefit of HOCl solution using Vashe® wound therapy as the principal wound solution for dressings in non-healing and malodorous wounds [11-12].

## Conclusions

While this is not the first description of the use of NPWT in the burn population, in our experience, the use of VVCC as an ROCF device with the use of HOCl irrigation results in a shorter time to wound closure using skin grafting techniques in the burn or necrotizing soft tissue infection patient populations.
